# Greater male variability in daily energy expenditure develops through puberty

**DOI:** 10.1098/rsbl.2023.0152

**Published:** 2023-09-20

**Authors:** Lewis G. Halsey, Vincent Careau, Philip N. Ainslie, Heliodoro Alemán-Mateo, Lene F. Andersen, Liam J. Anderson, Leonore Arab, Issad Baddou, Linda Bandini, Kweku Bedu-Addo, Ellen E. Blaak, Stephane Blanc, Alberto G. Bonomi, Carlijn V. C. Bouten, Pascal Bovet, Soren Brage, Maciej S. Buchowski, Nancy F. Butte, Stephan G. Camps, Regina Casper, Graeme L. Close, Lisa H. Colbert, Jamie A. Cooper, Richard Cooper, Prasangi Dabare, Sai Krupa Das, Peter S. W. Davies, Sanjoy Deb, Christine Delisle Nyström, William Dietz, Lara R. Dugas, Simon Eaton, Ulf Ekelund, Asmaa El Hamdouchi, Sonja Entringer, Terrence Forrester, Barry W. Fudge, Melanie Gillingham, Annelies H. Goris, Michael Gurven, Hinke Haisma, Catherine Hambly, Daniel J. Hoffman, Marije B. Hoos, Sumei Hu, Noorjehan Joonas, Annemiek Joosen, Peter Katzmarzyk, Kitty P. Kempen, Misaka Kimura, William E. Kraus, Wantanee Kriengsinyos, Rebecca Kuriyan, Robert F. Kushner, Estelle V. Lambert, Pulani Lanerolle, Christel L. Larsson, Nader Lessan, Marie Löf, Corby K. Martin, Eric Matsiko, Gerwin A. Meijer, James C. Morehen, James P. Morton, Aviva Must, Marian Neuhouser, Theresa A. Nicklas, Robert M. Ojiambo, Kirsi H. Pietilainen, Yannis P. Pitsiladis, Jacob Plange-Rhule, Guy Plasqui, Ross L. Prentice, Roberto Rabinovich, Susan B. Racette, David A. Raichen, Eric Ravussin, Leanne Redman, John J. Reilly, Rebecca M. Reynolds, Susan Roberts, Dulani Samaranayake, Luís B. Sardinha, Albertine J. Schuit, Analiza M. Silva, Srishti Sinha, Anders M. Sjödin, Eric Stice, Albert Stunkard, Samuel S. Urlacher, Mauro Eduardo Valencia, Giulio Valenti, Ludo M. van Etten, Edgar A. Van Mil, Jeanine A. Verbunt, Jonathan C. K. Wells, George Wilson, Brian Wood, Tsukasa Yoshida, Xueying Zhang, Alexia Murphy-Alford, Cornelia Loechl, Amy Luke, Herman Pontzer, Jennifer Rood, Hiroyuki Sagayama, Klaas R. Westerterp, William W. Wong, Yosuke Yamada, John R. Speakman, IAEA DLW Database Consortium

**Affiliations:** ^1^ School of Life and Health Sciences, University of Roehampton, London SW15 4JD, UK; ^2^ Department of Biology, University of Ottawa, Ottawa, Ontario, Canada; ^3^ Research Institute for Sport & Exercise Sciences, Liverpool John Moores University, Liverpool, L3 3AF, UK; ^4^ Coordinación de Nutrición, Centro de Investigación en Alimentación y Desarrollo (CIAD), A.C., Carretera Gustavo Enrique Astiazarán Rosas, No. 46, Col. La Victoria, C.P. 83304, Hermosillo, Sonora, México; ^5^ Department of Nutrition, Institute of Basic Medical Sciences, University of Oslo, 0317 Oslo, Norway; ^6^ School of Sport, Exercise and Rehabilitation Sciences, University of Birmingham, Birmingham, B15 2TT, UK; ^7^ David Geffen School of Medicine, University of California, Los Angeles, CA, USA; ^8^ Unité Mixte de Recherche en Nutrition et Alimentation, CNESTEN-Université Ibn Tofail, Rabat, PC.10100, Morocco; ^9^ University of Massachusetts Chan Medical School, Worcester, MA, USA; ^10^ Department of Physiology, Kwame Nkrumah University of Science and Technology, Kumasi, Ghana; ^11^ Department of Human Biology, Nutrition and Translational Research in Metabolism (NUTRIM), Maastricht University Medical Centre, Maastricht, 6200 MD, Netherlands; ^12^ Institut Pluridisciplinaire Hubert Curien, CNRS Université de Strasbourg, Strasbourg, France; ^13^ Phillips Research, Eindhoven, The Netherlands; ^14^ Department of Biomedical Engineering and Institute for Complex Molecular Systems, Eindhoven Unversity of Technology, Eindhoven, The Netherlands; ^15^ University Center for primary care and public health (Unisante), 1012 Lausanne, Switzerland; ^16^ MRC Epidemiology Unit, University of Cambridge, Cambridge, UK; ^17^ Division of Gastroenterology, Hepatology and Nutrition, Department of Medicine, Vanderbilt University, Nashville, TN, USA; ^18^ Department of Pediatrics, Baylor College of Medicine, USDA/ARS Children's Nutrition Research Center, Houston, TX, 77030, USA; ^19^ imec within OnePlanet Research Center, 6708 WE, Wageningen, The Netherlands; ^20^ Stanford University School of Medicine, Department of Psychiatry, Stanford, CA 94305, USA; ^21^ Kinesiology, University of Wisconsin, Madison, WI, USA; ^22^ Nutritional Sciences, University of Wisconsin, USA; ^23^ Department of Public Health Sciences, Parkinson School of Health Sciences and Public Health, Loyola University Chicago, Maywood, IL 60153, USA; ^24^ Department of Physiotherapy, Faculty of Allied Health Sciences, General Sir John Kotelawala Defence University, Sri Lanka; ^25^ Jean Mayer USDA Human Nutrition Research Center on Aging, Tufts University, Boston, MA, 02111, USA; ^26^ Child Health Research Centre, Level 6 Centre for Children's Health Research, University of Queensland, 62 Graham Street, South Brisbane, Queensland, 4101, Australia; ^27^ Centre for Nutraceuticals, School of Life Sciences, University of Westminster, London, UK; ^28^ Department of Circulation and Medical Imaging, Faculty of Medicine and Health Sciences, Norwegian University of Science and Technology, Trondheim, Norway; ^29^ Department of Biosciences and Nutrition, Karolinska Institutet, 141 83 Huddinge, Stockholm, Sweden; ^30^ George Washington University, Washington, DC, USA; ^31^ Division of Epidemiology and Biostatistics, School of Public Health, University of Cape Town, Cape Town, South Africa; ^32^ UCL Great Ormond Street Institute of Child Health, London, WC1N 1EH, UK; ^33^ Department of Sport Medicine, Norwegian School of Sport Sciences, PO Box 4014, 0806 Ulleval Stadion, Oslo, Norway; ^34^ Charité – Universitätsmedizin Berlin, corporate member of Freie Universität Berlin, Humboldt-Universität zu Berlin, and Berlin Institute of Health (BIH), Institute of Medical Psychology, Berlin, Germany; ^35^ University of California Irvine, Irvine, CA, USA; ^36^ Solutions for Developing Countries, University of the West Indies, Mona, Kingston, Jamaica; ^37^ Physiology Department, Aspire Academy, Doha, PO Box 22287, Qatar; ^38^ Department of Molecular and Medical Genetics, Oregon Health & Science University, Portland, OR 97239, USA; ^39^ Department of Anthropology, University of California Santa Barbara, Santa Barbara, CA 93106, USA; ^40^ Population Research Centre, Faculty of Spatial Sciences, Landleven 1, 9747AD, University of Groningen, Groningen, Netherlands; ^41^ Institute of Biological and Environmental Sciences, University of Aberdeen, Aberdeen, AB24 2TZ, UK; ^42^ Department of Nutritional Sciences, Program in International Nutrition, Rutgers University, New Brunswick, NJ 08901 USA; ^43^ Institute of Genetics and development Biology, Chinese Academy of Sciences, Beichen Xi lu, Beijing, People's Republic of China; ^44^ Central health Laboratory, Ministry of Health and Wellness, Port Louis, 72259, Mauritius; ^45^ Pennington Biomedical Research Center, Baton Rouge, LA, 70808, USA; ^46^ Institute for Active Health, Kyoto University of Advanced Science, Kyoto, Japan; ^47^ Department of Medicine, Duke University, Durham NC 27708, USA; ^48^ Dept. of Evolutionary Anthropology, Duke University, Durham NC 27708, USA; ^49^ Duke Global Health Institute, Duke University, Durham NC 27708, USA; ^50^ Institute of Nutrition, Mahidol University, Salaya, Thailand; ^51^ Division of Nutrition, St John's Research Institute, Bangalore, Karnataka - 560034, India; ^52^ Northwestern University, Chicago, IL, USA; ^53^ Health through Physical Activity, Lifestyle and Sport Research Centre, Division of Exercise Science and Sports Medicine (ESSM), FIMS International Collaborating Centre of Sports Medicine, Department of Human Biology, Faculty of Health Sciences, University of Cape Town, Cape Town, South Africa; ^54^ Department of Biochemistry and Molecular Biology, Faculty of Medicine, University of Colombo, Colombo, Sri Lanka; ^55^ Department of Food and Nutrition, and Sport Science, University of Gothenburg, Gothenburg SE-405 30, Sweden; ^56^ Imperial College London Diabetes Centre, Abu Dhabi, United Arab Emirates; ^57^ Department of Human Nutrition and Dietetics, University of Rwanda, Kigali, Rwanda; ^58^ Tufts University School of Medicine, Boston, USA; ^59^ Division of Public Health Sciences, Fred Hutchinson Cancer Center and School of Public Health, University of Washington, Seattle, WA, 98109, USA; ^60^ Moi University, Eldoret, Kenya; ^61^ University of Global Health Equity, Rwanda; ^62^ Helsinki University Central Hospital, Helsinki, Finland; ^63^ University of Brighton, Eastbourne, UK; ^64^ Department of Nutrition and Movement Sciences, Maastricht University, 6200 MD Maastricht, The Netherlands; ^65^ University of Edinburgh, Edinburgh, UK; ^66^ College of Health Solutions, Arizona State University, Phoenix, AZ, 85004, USA; ^67^ Biological Sciences and Anthropology, University of Southern California, CA, USA; ^68^ Department of Psychological Sciences and Health, University of Strathclyde, Glasgow, UK; ^69^ Centre for Cardiovascular Sciences, Queen's Medical Research Institute, University of Edinburgh, Edinburgh, EH16 4TJ, UK; ^70^ Department of Community Medicine, Faculty of Medicine, University of Colombo, Colombo, Sri Lanka; ^71^ Exercise and health laboratory, CIPER, Faculdade Motricidade Humana, Universidade de Lisboa, Portugal; ^72^ Executive Board, Tilburg University, Tilburg, Noord-Brabant, 5037 AB, The Netherlands; ^73^ Department of Nutrition, Exercise and Sports, Copenhagen University, Copenhagen, Denmark; ^74^ PhD Department of Psychiatry and Behavioral Sciences, Stanford University, 401 Quarry Road, Stanford, CA 94305; ^75^ University of Pennsylvania Perelman School of Medicine Department of Psychiatry; ^76^ Department of Anthropology, Baylor University, Waco, TX 76706, USA; ^77^ Chair Youth, Food and Health, Maastricht University, 5911 BV, Venlo, and Lifestyle Medicine Center for Children, Jeroen Bosch Hospital 5223 GW `s-Hertogenbosch, The Netherlands; ^78^ Population, Policy and Practice Research and Teaching Department, UCL Great Ormond Street Institute of Child Health, London, WC1N 1EH, UK; ^79^ University of California Los Angeles, Los Angeles, 90095, USA; ^80^ Department of Human Behavior, Ecology, and Culture, Max Planck Institute for Evolutionary Anthropology, Leipzig 04103, Germany; ^81^ National Institute of Health and Nutrition, National Institutes of Biomedical Innovation, Health and Nutrition, Tokyo, Japan; ^82^ Shenzhen Key Laboratory of Metabolic Health, Center for Energy Metabolism and Reproduction, Shenzhen Institute of Advanced Technology, Chinese Academy of Sciences, Shenzhen, 518055, China; ^83^ Nutritional and Health Related Environmental Studies Section, Division of Human Health, International Atomic Energy Agency, Vienna, Austria; ^84^ Faculty of Health and Sport Sciences, University of Tsukuba, Ibaraki, 305-8574, Japan

**Keywords:** inter-individual variation, morphometry, age, height, weight

## Abstract

There is considerably greater variation in metabolic rates between men than between women, in terms of basal, activity and total (daily) energy expenditure (EE). One possible explanation is that EE is associated with male sexual characteristics (which are known to vary more than other traits) such as musculature and athletic capacity. Such traits might be predicted to be most prominent during periods of adolescence and young adulthood, when sexual behaviour develops and peaks. We tested this hypothesis on a large dataset by comparing the amount of male variation and female variation in total EE, activity EE and basal EE, at different life stages, along with several morphological traits: height, fat free mass and fat mass. Total EE, and to some degree also activity EE, exhibit considerable greater male variation (GMV) in young adults, and then a decrease in the degree of GMV in progressively older individuals. Arguably, basal EE, and also morphometrics, do not exhibit this pattern. These findings suggest that single male sexual characteristics may not exhibit peak GMV in young adulthood, however total and perhaps also activity EE, associated with many morphological and physiological traits combined, do exhibit GMV most prominently during the reproductive life stages.

## 1. Introduction

Individuals of a sexually reproducing species vary in terms of nearly every measurable characteristic, morphological, physiological and cognitive. In mammals, often the magnitude of this inter-individual variability, at least in terms of body morphometrics and, in humans, also cognition, has been reported as greater between males than between females [1,2]. This phenomenon is termed ‘greater male variability’ (GMV) [3]. For example, it has been reported that human males are more varied in their physical performance than are human females [4,5], and male chimpanzees have more varied brain structures than do female chimpanzees [6]. Given that the metabolism of an animal is arguably an emergent property influenced by the culmination of GMV in various bodily traits, in a previous study we postulated that GMV in energy expenditure could be particularly large [7]. Supporting this suggestion, we found that in adult humans, energy expenditure exhibits considerable GMV in terms of basal energy expenditure (BEE) and total (daily) expenditures (TEE), even after controlling for key morphological traits such as height, fat free mass and fat mass [7].

We also discovered that with ageing, variation between people in their TEE decreases, but this happens more rapidly in men than in women which results in the magnitude of GMV in TEE attenuating in older age groups [7]. One possible explanation for GMV is that males experiencing stronger sexual selection results in the expression of male sexual traits with greater variance than that of female sexual traits [8]. Energy expenditure is related to various traits of sexual interest to females such as cognitive capacity [9], physical endeavour, strength and muscle mass [10], and perhaps also aerobic fitness [11]. Variability in male sexual characteristics could be most prominent during the period of life associated with sexual reproduction (i.e. late adolescence and young adulthood), explaining the aforementioned decrease in GMV in energy expenditure in older people.

If indeed GMV in energy expenditure is underpinned by sexual selection, we predict there will be no GMV in humans prior to sexual maturity, and that GMV will peak during young adulthood due to an increase in male variation, which will then decrease more rapidly than female variation into old age. We tested this hypothesis by analysing extensive data on human energy expenditure for individuals of ages spanning the entire human life course.

## 2. Methods

We analysed data from the International Atomic Energy Agency (IAEA) DLW database v.3.7 [12]. The dataset comprised TEE measurements (MJ d^−1^) for 4992 females and 2626 males, and BEE measurements (MJ d^−1^) using indirect calorimetry for 1542 females and 934 males. Estimates of activity energy expenditure (AEE) were calculated by subtracting BEE from 0.9*TEE (TEE adjusted to account for the thermic effect to food). Ages of the participants ranged from newborns to 101 years old. We also included in our analyses the following traits: height (cm), fat-free mass (kg), fat mass (kg) and body weight (kg). Further details are provided in Halsey *et al*. [7].

### (a) Statistical analyses

All analyses were conducted in R v. 3.5.3 [13]. We quantified variance in male and female energy expenditure using Bayesian general linear models based on Monte-Carlo Markov chain (MCMC) models using the ‘MCMCglmm’ package [14]. Each model included one trait as the response variable (TEE, AEE or BEE) and sex as an independent variable along with age as a categorical variable, and height, fat-free mass and fat mass as continuous variables, with no intercept fitted so that the model returned separate mean estimates for males and females within each age category [15]. Age was recoded as a categorical variable with eight levels for the TEE model, and six levels for the AEE and BEE models (due to a smaller overall BEE sample size, although sample size per age category was still smaller). Using the ‘idh’ function, we allowed the residual variance to be different in males and females within each age category. Further models included height, fat-free mass or fat mass as the response variable (with only sex and age as independent variables). All models also included country as a random effect to account for the unequal sampling distribution across countries. When included as covariates, the three morphometric variables were centred so that model estimates were estimated at the centre of the distribution of the covariates [15]. To make sure that any sex differences in variance were not due to mean differences, we also standardized the variance within each sex and age category by dividing variances with the sex-specific mean estimates to obtain the coefficient of variance (CoV). From each model, we calculated the posterior mode and 95% highest posterior density credible intervals (CIs) for the CoV estimates for each age group in males and females. Treating these as 95% confidence intervals, in cases where the CIs for the relative variances of the two sexes do not overlap, the evidence of a difference in variance between the sexes is strong [16]. Finally, we calculated the male : female ratio of CoV whereby a value greater than 1 indicated GMV.

## 3. Results

**Figure 1.**
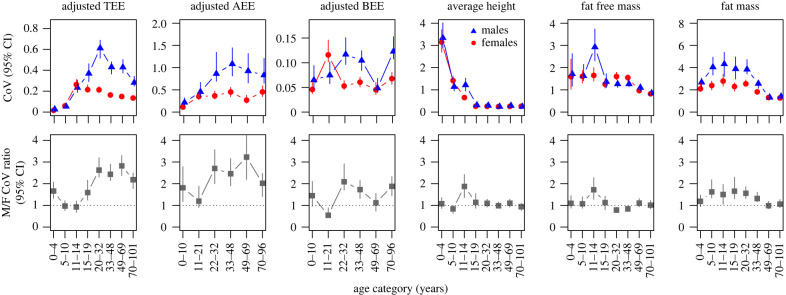
Top row: male (blue triangles) and female (red circles) coefficients of variance (CoV) in measures of energy expenditure and body morphometrics as a function of age category. Error bars represent 95% credible intervals (95% CI). These data are presented in electronic supplementary material, table S1. Bottom row: the ratio of male to female (M/F) CoV with 95% CI. The dashed lines represent equal variance; values above indicate greater male variance (GMV). TEE = total energy expenditure; AEE = activity energy expenditure; BEE = basal energy expenditure.

Visual interpretation of graphs of energy expenditure and morphology against age category (figure 1), using the 95% CIs for guidance, indicates that TEE and AEE exhibit increases in male variance from early childhood into young adulthood followed by decreases with further ageing. In TEE, this generates the development of GMV into young adulthood and a decrease in the elderly, while in AEE a pattern of increase and then decrease is apparent but less clear. A pattern in the data for BEE is not apparent although GMV does peak in early adulthood. Average height shows no pattern of GMV developing into young adulthood and then fading. Fat free mass also shows no such pattern. Fat mass does exhibit an increase in male variance followed by a decrease, which results in a similar pattern in GMV, however peak GMV occurs well before adulthood.

## 4. Discussion

In young children, there is little difference between boys and girls in terms of between-person variance in TEE (figure 1). However, by late adolescence (15–19 years) males are exhibiting greater variation and in turn GMV. This GMV increases in magnitude into young adulthood and beyond, and then somewhat decreases by old age due to decreasing inter-male variation. This pattern through the life course bears hallmarks of a sexually selected signal, or at least a trait that is an emergent property of characteristics some or all of which are sexually selected. The qualitative pattern of increasing then decreasing inter-male variation is also apparent although less strong in AEE, and increasing then decreasing GMV is also apparent. Albeit the sample size was lower and inter-study measurement variation probably higher, the above findings together tentatively suggest that changes in AEE variance over the life course might somewhat drive the TEE variance patterns.

Patterns for BEE are less clear and might be due to, again, a lower sample size than for TEE and with greater measurement variation between studies. More data are required.

Given that body morphology is considered to harbour sexual traits, we might predict that at least some morphological measures would exhibit a pattern of GMV similar to that seen in TEE, however this was not the case. While fat mass exhibits an increase then decrease in inter-male variation over the life course, the start of peak GMV occurs before adolescence rather than during the age of peak reproduction. Average height and fat free mass both show a spike in inter-male variation, and in turn substantive GMV, for a short period in early adolescence, an observation also apparent in data for Dutch children [17]. However, this is likely generated by the variability in both magnitude and degree of growth spurt in boys at this age.

About two-thirds of the dataset are for citizens of the USA, a country that has particularly high rates of obesity (https://www.oecd.org/health/health-data.htm). Nonetheless, qualitatively the patterns in male variation, female variation and GMV through the life course are generally similar to those present in the full dataset when the USA data are removed (electronic supplementary material, figure S1 and table S2).

In conclusion, inter-individual variance over the life course for height and fat free mass do not exhibit the hypothesized patterns given that they are two morphometric variables considered to be male sexual characteristics (albeit height is a relatively fixed variable come adulthood). Fat mass is the morphometric variable that is closest to exhibiting the hypothesized patterns, despite being considered more of a female sexual characteristic. TEE and to some extent AEE present with GMV developing and peaking in young adulthood and subsequently declining, perhaps indicating that they are either male sexual characteristics or related to such characteristics. Perhaps energy expenditure most clearly presents with the hypothesized patterns of GMV over the life course because it results from the combined effects of many characteristics.

## Data Availability

The data are provided in the electronic supplementary material [18].
