# Correction: ‘Greater male variability in daily energy expenditure develops through puberty’ (2023), by Halsey *et al.*

**DOI:** 10.1098/rsbl.2023.0482

**Published:** 2023-11-01

**Authors:** Biology Letters

**Keywords:** inter-individual variation, morphometry, age, height, weight

*Biol. Lett.* **19**, 20230152 (Published online 20 September 2023). (https://doi.org/10.1098/rsbl.2023.0152)

Since initial publication, changes have been made to the author list to correct author names, affiliations and general spelling on the Publisher's website. This does not affect the results of the paper. The list of affiliations can be found in the electronic supplementary material [1].
